# Estimating the impact of the COVID-19 pandemic on infectious disease notifications in Klang district, Malaysia, 2020–2022

**DOI:** 10.5365/wpsar.2025.16.01.1097

**Published:** 2025-01-27

**Authors:** Vivek Jason Jayaraj, Diane Woei-Quan Chong, Faridah Binti Jafri, Nur Adibah Binti Mat Saruan, Gurpreet Kaur Karpal Singh, Ravikanth Perumal, Shakirah Binti Jamaludin, Juvina Binti Mohd Janurudin, Siti Rohana Binti Saad

**Affiliations:** a Sector for Biostatistics and Data Repository, National Institutes of Health, Ministry of Health, Putrajaya, Malaysia.; b Institute for Health Systems Research, National Institutes of Health, Ministry of Health, Putrajaya, Malaysia.; c Klang District Health Office, Ministry of Health, Putrajaya, Malaysia.

## Abstract

**Objective:**

The COVID-19 pandemic disrupted disease surveillance systems globally, leading to reduced notifications of other infectious diseases. This study aims to estimate the impact of the COVID-19 pandemic on the infectious disease surveillance system in Klang district, Selangor state, Malaysia.

**Methods:**

Data on notifiable diseases from 2014 to 2022 were sourced from the Klang District Health Office. The 11 diseases with more than 100 notifications each were included in the study. For these 11 diseases, a negative binomial regression model was used to explore the effect of the pandemic on case notifications and registrations by year, and a quasi-Poisson regression model was used to explore the changes by week.

**Results:**

The results showed a reduction in the number of notifications and registrations for all 11 diseases combined during the pandemic compared with previous years. Changes between expected and observed notifications by week were heterogeneous across the diseases.

**Discussion:**

These findings suggest that restrictive public health and social measures in Klang district may have impacted the transmission of other infectious diseases during the COVID-19 pandemic. The differential impact of the pandemic on disease notifications and reporting highlights the large ancillary effects of restrictive public health and social measures and the importance of building resilience into infectious disease surveillance systems.

The COVID-19 pandemic significantly impacted global disease surveillance, leading to disruptions within disease notification systems. The pandemic significantly strained health systems, resulting in reduced capacities for notifications and case detection. (*1*) However, the restrictions on mobility due to public health and social measures resulted in reduced transmission of infectious diseases within the community.

Malaysia, along with numerous other nations, was severely affected by the COVID-19 pandemic. As of March 2023, Malaysia had reported more than 4 million COVID-19 cases and more than 45 000 deaths. (*2*) The pandemic prompted the implementation of various public health and social measures, such as movement restrictions, border controls and mask mandates. These interventions were effective in reducing COVID-19 transmission within the community. (*3*, *4*) However, public health and social measures have had other well documented, ancillary effects on societies and economies. (*5*)

A surveillance system in public health refers to the continuous and systematic collection, analysis and interpretation of health-related data that are essential for planning, implementing and evaluating public health practices. (*6*) These systems play a pivotal role in detecting, monitoring and responding to emerging and endemic diseases to safeguard global health security. (*7*) Malaysia has an established infectious disease notification system, with all health-care providers mandated by law to report cases to the Ministry of Health. (*8*) The reduction in human mobility and the strain on the health-care system during the COVID-19 pandemic may have had unintended effects on the notification of other infectious diseases. (*1*, *9*) However, quantifying the effect of acute shocks on a surveillance system can allow for the necessary calibration of a system's capabilities, both in the short term and for future readiness. The objective of this study was to investigate the impact of the COVID-19 pandemic on the surveillance of infectious diseases in Klang district, Selangor state, Malaysia from 2020 to 2022.

## Methods

### Study setting, data source and inclusion criteria

The Klang District Health Office serves a population of 1.1 million over a land area of 6 km^2^, making it one of the most densely populated areas served by a district health office in Selangor. (*10*) In Malaysia, the Prevention and Control of Infectious Diseases Act 1988 (Act 342) mandates the reporting of 31 diseases by health-care facilities. (*8*) All diseases included in the Act are notified, verified and registered via an e-notification system except for dengue, measles, tuberculosis (TB) and HIV, which are handled by integrated ancillary systems for case registration and management.

Notification refers to the mandatory reporting of specific infectious diseases by health-care providers to relevant health authorities upon diagnosis, whether suspected or confirmed. Notifications are then verified to determine whether they meet the case definition before a patient is registered as a confirmed case. The verification process conducted by public health inspectors – involving phone checks and source tracing to confirm the authenticity and accuracy of notifications – experienced delays during the COVID-19 pandemic due to resource diversion. Verified notifications meeting the case definition for a disease are registered. Batch processing of registrations – whereby notifications are not registered immediately but might instead be cumulatively registered, for instance, at the end of a week – can lead to discrepancies in the data, showing more registrations than notifications in certain weeks. This does not necessarily indicate a true change in disease patterns but rather reflects the timing of data entry. The case definition and clinical and laboratory criteria are based on Malaysia’s case definition guidelines, (*11*) and these did not undergo any major revision for the diseases studied during the study period.

Data on all notifiable diseases were sourced from the Klang District Health Office between epidemiological week 1 of 2014 (29 December 2013) and epidemiological week 52 of 2022 (31 December 2022). Age, sex, date of notification and date of registration were extracted for each notification. Diseases with fewer than 100 notifications or registrations per year were excluded from the analysis because these small counts are less likely to be accurately modelled (i.e. chikungunya, cholera, leprosy, malaria, rabies, tetanus, typhus, typhoid/paratyphoid, pertussis and viral encephalitis). Notifiable diseases included in the analysis were dengue; leptospirosis; foodborne illness; dysentery; measles; hand, foot and mouth disease (HFMD); TB; HIV; gonorrhoea; syphilis; and viral hepatitis. Additionally, data about policies were obtained from the Oxford COVID-19 Government Response Tracker, (*12*) specifically the Stringency Index and information about school closures, which quantify the strictness of government measures and the status of educational institutions, respectively.

### Data analysis

Data were collated and aggregated into weekly notification and registration counts for all diseases and for each disease separately. Two analyses were conducted using different dimensions of data and statistical approaches, as previously described. (*1*, *9*) The first analysis examined the effect of the COVID-19 pandemic on case notifications and registrations in Klang district.

The between-period rate of change in case notifications and registrations was estimated using a negative binomial regression model. The model estimated the effect of the pandemic on notifications and registrations by comparing baseline (2014–2019) and pandemic years (2020–2022). Exponentiated coefficients from the model represent the multiplicative effect on notifications and registrations, quantifying increases or decreases relative to the baseline. The regression coefficient (*β*) used in estimating the percentage change in notifications and registrations (1 – exp (*β*) across the different periods is given by the function:

log(E(count)) = α+β1(pandemic)

where E(count) is the expected count, α is the intercept and β1 is the effect of the pandemic variable on the log count.

The second analysis estimated and was used to visualize the weekly change in the frequency of case notifications and registrations. A quasi-Poisson regression model was trained using observed count data from week 1 of 2014 to week 52 of 2019. The model included terms for trend and seasonality. This model is given by the function:

where α is the intercept, β1 is the effect of the pandemic variable on the log count, β2 is the linear time trend and β3 is the seasonal variation based on the week of the year.

This baseline model was used to predict the expected counts of notifications and registrations from week 1 of 2020 to week 52 of 2022. The expected counts were then compared with the observed counts, and a weekly rate of change was estimated and visualized. The 95% confidence intervals (95% CIs) were estimated through bootstrapping. Goodness-of-fit of the models was assessed using the Akaike information criterion and log-likelihood values. Data analyses were performed with R software v. 4.3.2 (R Core Team, Vienna, Austria) using the tidyverse and MASS packages. (*13*)

## Results

**Fig. 1:**
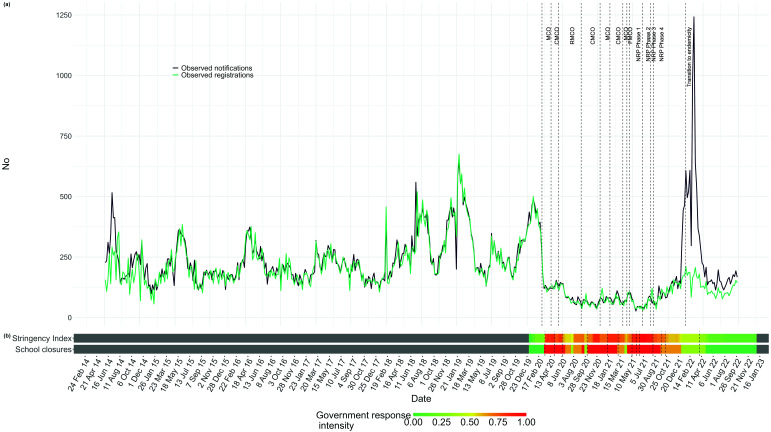
(a) All notifications and registrations for the 11 diseases included in the study,a Klang district, Malaysia, 2014–2022; (b) COVID-19 government response intensity, Malaysia, 2020–2022

**Fig. 2:**
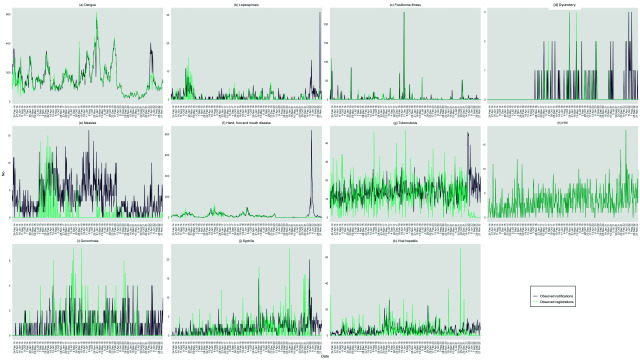
No. of notifications and registrations, by disease, Klang district, Malaysia, 2014–2022a

Relative to the pre-pandemic period, the total number of notifications and registrations decreased in 2020 and 2021 in Klang district before increasing again from mid-2022 onwards. Throughout the pandemic, variations in the stringency of policies, particularly during periods of increased restrictions and school closures, were observed to correlate with fluctuations in notifications and registrations. Notably, spikes in notifications and registrations often followed the relaxation of these measures (**Fig. 1**). This increase was mostly due to notifications of HFMD and dengue (**Fig. 2**). In late 2022, weekly registrations were lower than the number of notifications for HFMD, dengue, TB, gonorrhoea, syphilis, viral hepatitis and leptospirosis (**Fig. 2**).

[**insert** Figure 1]

[**insert** Figure 2]

In the first analysis, the number of notifications and registrations for all 11 diseases combined in 2020 compared with the reference period of 2014–2019 changed by −38% (95% CI: −46% to −30%) for notifications and −37% (95% CI: −44% to −29%) for registrations. Further declines for both categories were observed in 2021: −69% (95% CI: −73% to −65%) for notifications and −69% (95% CI: −72% to −65%) for registrations. In 2022, notifications were 8% higher (95% CI: −4% to 22%) than during the reference period of 2014–2019, while case registrations were −43% (95% CI: −49% to −36%) (**Table 1**).

**Table 1:** Differences in notifications and registrations, by year, 2020–2022 compared with 2014–2019, Klang district, Malaysia

Disease	Notifications^a^	Registrations^a^
**All diseases**		
**2014–2019**	**Reference**	**Reference**
**2020**	**-38 (−46, −30)**	**-37 (−44, −29)**
**2021**	**-69 (−73, −65)**	**-69 (−72, −65)**
**2022**	**8 (−4, 22)**	**-43 (−49, −36)**
**Dengue**		
**2014–2019**	**Reference**	**Reference**
**2020**	**-38 (−46, −29)**	**-36 (−44, −27)**
**2021**	**-76 (−79, −72)**	**-75 (−78, −71)**
**2022**	**-20 (−30, −8)**	**-34 (−42, −24)**
**Leptospirosis**		
**2014–2019**	**Reference**	**Reference**
**2020**	**-41 (−64, −5)**	**-52 (−79, 16)**
**2021**	**-72 (−85, −50)**	**-70 (−88, −25)**
**2022**	**194 (105, 330)**	**-69 (−88, −23)**
**Foodborne illness**		
**2014–2019**	**Reference**	**Reference**
**2020**	**-85 (−93, −62)**	**-92 (−98, −63)**
**2021**	**-24 (−65, 85)**	**-35 (−82, 205)**
**2022**	**-58 (−80, 0)**	**-97 (−99, −83)**
**Dysentery**		
**2014–2019**	**Reference**	**Reference**
**2020**	**57 (−28, 218)**	**58 (−23, 196)**
**2021**	**14 (−53, 148)**	**15 (−50, 131)**
**2022**	**273 (108, 565)**	**273 (126, 503)**
**Measles**		
**2014–2019**	**Reference**	**Reference**
**2020**	**-53 (−63, −40)**	**-72 (−90, −5)**
**2021**	**-77 (−83, −69)**	**-92 (−98, −66)**
**2022**	**-53 (−63, −41)**	**-80 (−94, −33)**
**Hand, foot and mouth disease**		
**2014–2019**	**Reference**	**Reference**
**2020**	**-84 (−88, −77)**	**-84 (−89, −77)**
**2021**	**-96 (−98, −94)**	**-97 (−98, −95)**
**2022**	**196 (121, 306)**	**-93 (−95, −89)**
**Tuberculosis**		
**2014–2019**	**Reference**	**Reference**
**2020**	**6 (−4, 18)**	**5 (−16, 31)**
**2021**	**1 (−9, 12)**	**1 (−18, 26)**
**2022**	**46 (32, 61)**	**-76 (−81, −69)**
**HIV**		
**2014–2019**	**Reference**	**Reference**
**2020**	**16 (−7, 44)**	**16 (−7, 44)**
**2021**	**29 (4, 59)**	**29 (4, 59)**
**2022**	**71 (40, 109)**	**71 (40, 109)**
**Gonorrhoea**		
**2014–2019**	**Reference**	**Reference**
**2020**	**41 (1, 94)**	**41 (1, 94)**
**2021**	**-28 (−53, 7)**	**-28 (−53, 7)**
**2022**	**14 (−20, 60)**	**14 (−20, 60)**
**Syphilis**		
**2014–2019**	**Reference**	**Reference**
**2020**	**87 (45, 142)**	**112 (17, 311)**
**2021**	**45 (10, 91)**	**106 (14, 300)**
**2022**	**172 (114, 246)**	**-50 (−75, 6)**
**Viral hepatitis**		
**2014–2019**	**Reference**	**Reference**
**2020**	**2 (−20, 32)**	**-6 (−47, 76)**
**2021**	**-8 (−28, 19)**	**26 (−27, 136)**
**2022**	**37 (8, 75)**	**-75 (−87, −51)**

^a^ Values are% (95% confidence interval).

Comparisons of yearly notifications and registrations varied by disease (**Table 1**). Notifications of dengue, foodborne illness, leptospirosis, HFMD and measles decreased during 2020–2022, while those for TB, HIV, gonorrhoea, syphilis and viral hepatitis increased or remained static (**Table 1**).

**Fig. 3:**
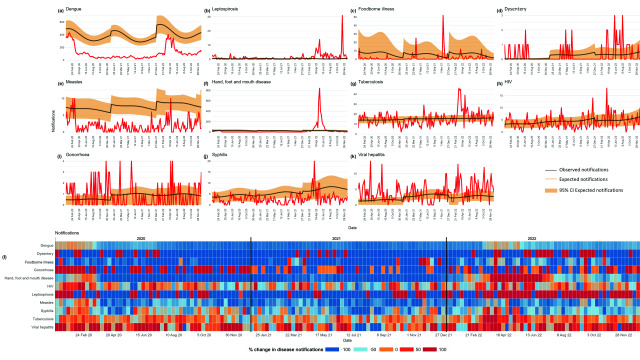
Differences in weekly notifications and percentage change between observed and expected notifications, by disease, Klang district, Malaysia, 2020–2022

In the second analysis, comparisons between weekly expected and observed notifications were heterogeneous across the diseases (**Fig. 3**). Dengue, measles and HFMD exhibited clear and consistent reductions in notifications in 2020 and 2021, followed by increases in 2022. However, other diseases, such as foodborne illness and dysentery, showed less consistent reductions.

[**insert** Figure 3]

The exponentiated coefficients for dengue, foodborne illness, dysentery, measles and HFMD indicate there was a decrease in case notifications of 100% or more in 2020 and 2021 when compared with the predicted point estimate, which then moved towards the baseline in week 5 of 2022. In 2022, there was a significant surge in notifications for leptospirosis, dysentery and HFMD, with observed rates substantially exceeding those projected by pre-pandemic trends. During 2020 and 2021, trends for TB, HIV and syphilis were similar to what was expected, with the rates of change for notifications increasing in 2022 in some weeks. Notifications for gonorrhoea and viral hepatitis showed increases of 100% for many weeks during 2020 and 2022, and to a lesser extent during 2021 (**Fig. 3**).

**Fig. 4:**
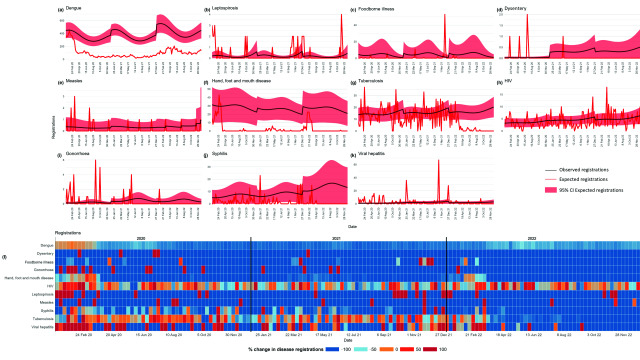
Differences in weekly registrations and percentage change between observed and expected registrations, by disease, Klang district, Malaysia, 2020–2022

Comparisons between weekly expected and observed registrations were less heterogeneous between diseases. All diseases except TB, HIV, syphilis and viral hepatitis had consistent reductions in the number of registrations between 2020 and 2022 compared with the reference period (**Fig. 4**). However, there were sporadic weeks in each year during which some diseases had 100% more registrations than expected, such as leptospirosis, measles and gonorrhoea (**Fig. 4**). The predicted number of notifications and registrations compared against the observed values are reported in **Supplementary Fig. 1** and **2**, respectively.



[**insert** Figure 4]

## Discussion

The COVID-19 pandemic has had a staggering impact on global health systems, which affected their ability to respond to other diseases. The results of this study showed a decline in disease notifications and registrations in 2020 and most of 2021 in Klang district, Malaysia, followed by increases in both between week 48 in 2021 and week 15 in 2022, compared with the reference period of 2014–2019. Since then, notifications and registrations have returned to near pre-pandemic levels for some, but not all, diseases.

The reductions in notifications and registrations for nearly all diseases across 2020 and 2021 were likely due to changes in interactions among people as a result of public health and social measures, which interrupted chains of transmission. Malaysia, in line with many other countries, introduced phased lockdowns and school closures during 2020 and 2021, intensifying these during each wave of COVID-19 and as variants emerged; these measures then eased as vaccinations were rolled out and policies shifted in early 2022. These types of interventions led to large reductions in the incidence of childhood diseases and foodborne and waterborne illnesses globally due to mobility restrictions. (*1*, *9*, *14*-*16*) Reductions in the incidence of childhood diseases, such as measles and HFMD, as well as foodborne illnesses and leptospirosis, in Klang district are consistent with these findings. The decline in mobility due to pandemic-related restrictions corresponded with decreased notifications of vector-borne diseases, such as dengue and malaria. These decreases occurred despite the likelihood that the vectors themselves remained unaffected by mobility restrictions. Similar patterns have been observed in other countries, (*17*, *18*) suggesting that this phenomenon is not unique to Malaysia but reflects a broader global trend.

Restrictive public health and social measures also likely modified the population’s health-seeking behaviour. Due to fear of contracting COVID-19 in health-care facilities, visits to providers were reduced, likely contributing to the decrease in notifications and registrations. (*1*, *19*) This has been reported to be an important factor affecting reductions in notifications of sexually transmitted infections in other settings. (*17*, *18*, *20*) Similarly, the number of notifications of respiratory illnesses that have longer latencies, such as TB, also fell significantly during the COVID-19 pandemic. (*21*)

In addition to public health and social measures, broader societal and environmental factors have been suggested as modifying disease dynamics during the pandemic. The widespread adoption of work-from-home practices and a substantial reduction in international travel likely altered traditional patterns of disease transmission, particularly for communicable diseases typically spread through close contact or travel. (*22*) Furthermore, environmental factors, such as urbanization and climate change, have been shown to affect disease vectors and transmission pathways, possibly also impacting the incidence of diseases during this period. (*23*)

The results of this study suggest that sexually transmitted infections, such as HIV and syphilis, as well as TB, which has a longer latency, had relatively constant reporting over time, indicating that surveillance systems for these diseases may have been less impacted by the pandemic than those for other diseases. Diseases with longer latency may have been less strongly impacted by acute shocks from changes in population mobility and behaviour. The health-care infrastructure for managing these diseases is often separate from acute care services, which may have shielded them from the resource redirection seen in other areas of health care during the pandemic. This separation could have enabled more consistent surveillance and reporting for these conditions. Consequently, surveillance programmes for these diseases maintained continuous reporting even during the pandemic.

Beginning in April 2022, there was a significant discrepancy between the number of notifications and the number of registrations during the time that public health and social measures were lifted completely in Malaysia. Much of this difference was driven by an increased circulation of diseases common in children, such as HFMD. The lifting of restrictions led to a large increase in notifications of HFMD during this period, and many of these were not subsequently registered as they did not meet the criteria for a confirmed case. This large increase in notifications signals a potential immunity-debt event, as has been described previously, whereby the reopening of schools led to the exposure of a large pool of immune-naive, susceptible children to diseases not actively circulating during the preceding 2 years. (*24*-*26*) However, this remains conjecture and requires further investigation. Conversely, discrepancies between notifications and registrations for TB, gonorrhoea, syphilis, viral hepatitis and leptospirosis in late 2022 were attributed to delays in laboratory confirmation, such as for testing repeat sputum samples for TB and the wait times for microagglutination testing results for leptospirosis, as opposed to actual changes in disease patterns.

Finally, it is crucial to recognize that the pandemic's influence on disease surveillance systems may have been exacerbated by conflicting priorities in an overburdened health system. This can lead to gaps in surveillance reporting so that certain cases or data points are missed or unrecorded for various reasons, as has been documented elsewhere. (*1*, *9*) However, while this study primarily focused on reductions in notifications and registrations, these may have been influenced by changes in population mobility and behaviours. This speculation aligns with findings from studies exploring the impact of changes in mobility and behaviour on disease dynamics during the pandemic. These studies have suggested that factors such as fear, perception of health risk, and cultural differences in mobility and social behaviour could have significantly impacted disease notification rates. (*1*, *18*, *27*-*29*) The studies also suggested that many of the changes in disease notification and registration trends can be explained by changes in public health and social measures. Nonetheless, further studies will be necessary to disentangle the role of local surveillance in driving these trends.

Our findings have several implications for disease surveillance and response activities. First, adopting newer technologies and more comprehensive surveillance strategies – including the use of mobile health applications, alternative data sources, crowd-sourced data, community surveillance and telemedicine – may better facilitate disease reporting and surveillance during crises. Second, if a pandemic response is prolonged, it may be necessary to continually update health-care facilities about the importance of vigilance for all infectious diseases within the community, especially when a community is transitioning into endemicity. Finally, analytical frameworks that can differentiate between genuine reductions in disease incidence and perceived reductions due to changes in societal behaviour or mobility patterns should be developed further. Understanding this distinction may enable systems to generate more accurate public health responses and strategies, even during public health emergencies such as pandemics.

This study had several limitations. These included model fits being affected by very few case numbers for certain diseases and time frames, and complexities in trend analysis. Other challenges included lag times for registrations and bulk entry of registrations, false-positive notifications causing artificial spikes in data (notably in HFMD cases) and discrepancies between weekly notifications and registrations due to delayed case verification. The COVID-19 response also extended verification times. These factors may influence data interpretation. Despite these limitations, we believe these models are still useful if interpreted carefully.

In conclusion, our study found decreases in disease notifications and registrations during the COVID-19 pandemic compared with pre-pandemic years. The differential impact of the pandemic on disease notification and reporting suggests that the restrictive public health and social measures implemented for COVID-19 impacted other diseases, although changes to the surveillance system during the pandemic may have also had effects. This highlights the importance of building resilience into infectious disease surveillance systems.
