# Supplementary material for: Estimating the impact of the COVID-19 pandemic on infectious disease notifications in Klang district, Malaysia, 2020–2022
Source: Western Pac Surveill Response J. 2025 Jan 27;16(1):1–9. doi: 10.5365/wpsar.2025.16.01.1097 (PMC11842894; doi:10.5365/wpsar.2025.16.01.1097)

Supplementary Fig. 1. **Predicted versus observed notifications, by disease, Klang district, Malaysia, 2014–2019**

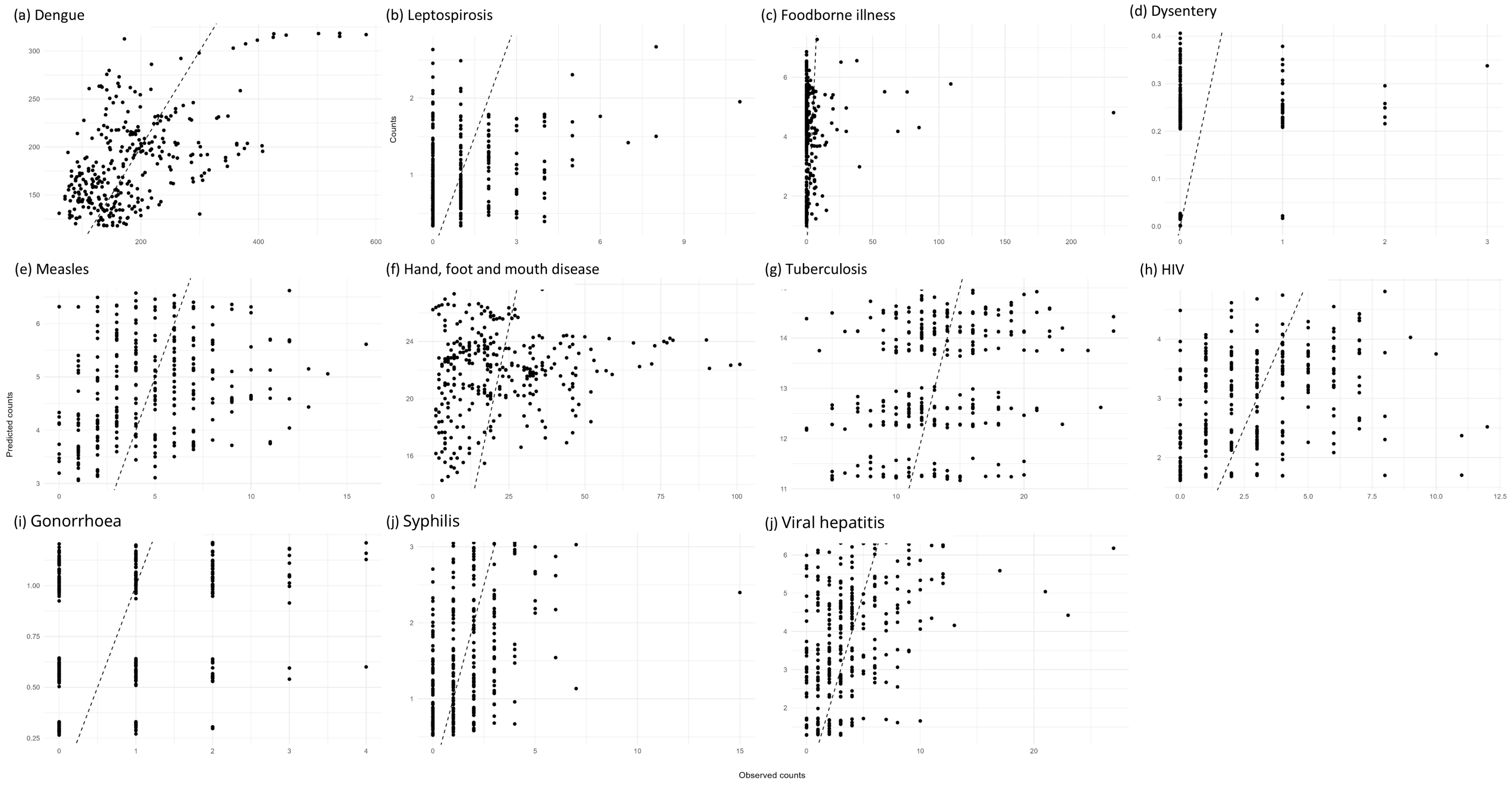

Supplementary Fig. 2. **Predicted versus observed registrations, by disease, Klang district, Malaysia, 2014–2019**

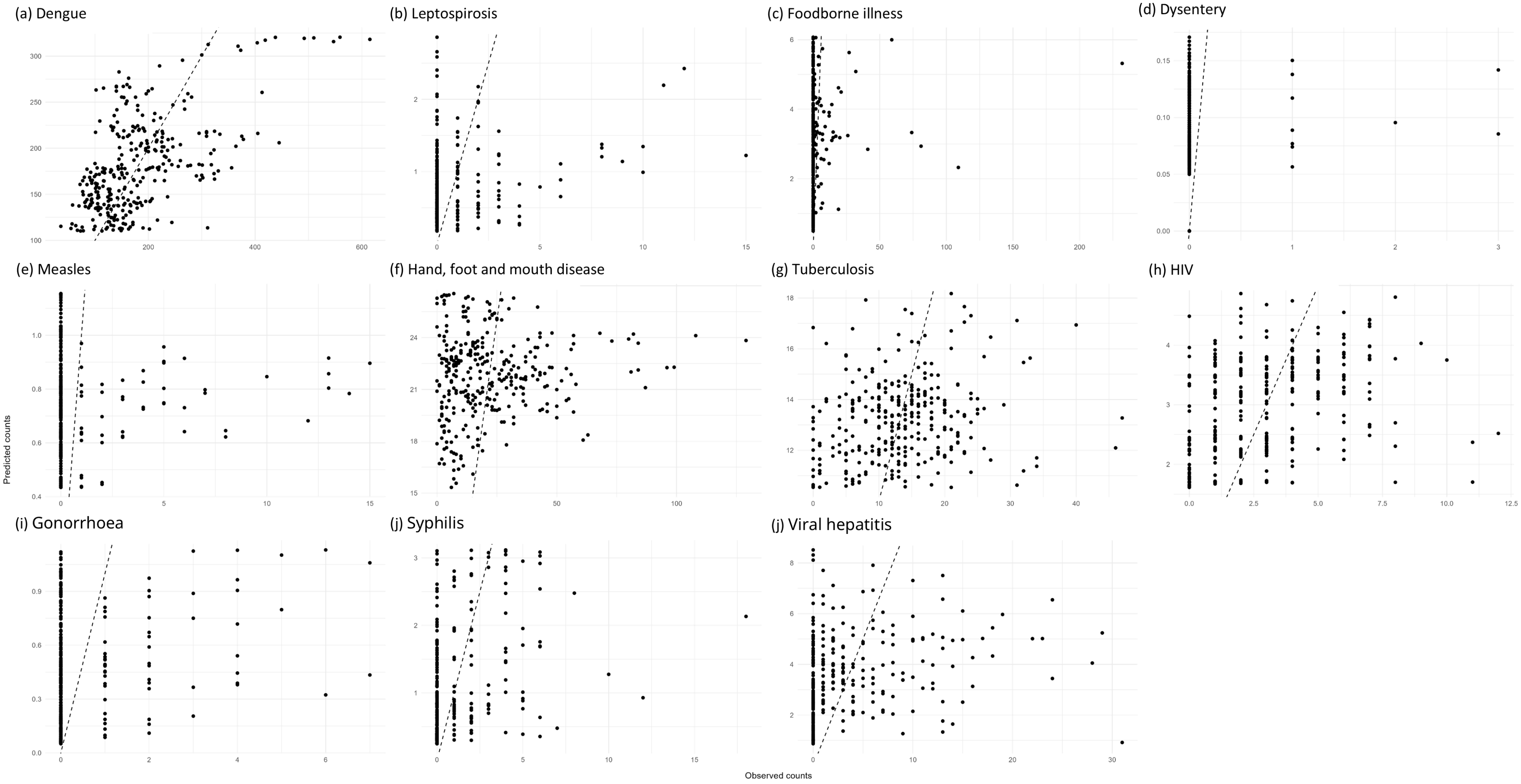

Supplement: Supplementary file 1 [file wpsar-16-1097-s001.pdf]
